# Unexpected difficult extubation of double lumen bronchial intubation: a case report

**DOI:** 10.1186/s12871-021-01512-5

**Published:** 2021-12-10

**Authors:** Xingcai Zhang, Shumiao Tang, Zihui Lu, Yijun Chen

**Affiliations:** Department of Anesthesiology, Ningbo City First Hospital, No. 59 Liuting Street, Haishu District, Ningbo, Zhejiang China

**Keywords:** Difficult extubation, Communication, VATS, Anaesthetic management

## Abstract

**Background:**

The anesthetist and other members of the perioperative team need to be extremely cautious for successful completion of any surgery. If the final step of any general anesthetic-extubation is not sufficiently well planned, it can lead to critical airway incidents during the extubation and hinder transportation of the patient to the post-anesthesia care unit.

**Case presentation:**

A 48-year-old female underwent video-assisted thoracoscopic surgery (VATS) combined with left lower lobectomy. The distal end of the left branch of the tracheal tube was lodged by surgical sutures. In this case, the respiratory physician burned the sutures using an argon electrode, after discussion with the thoracic surgery experts.

**Conclusions:**

Teamwork is essential when caring for a patient with a shared airway. The anesthetist and surgeon must communicate well with each other to achieve optimal surgical outcomes. Importantly, testing the patency of the ETT prior to extubation should be a regular procedure, which is practical significance to guide safe extubation.

## Background

Due to the COVID-19 epidemic, chest computed tomography (CT) has become a routine screening method for most admitted patients, which has caused increasing number of lung nodules to be discovered. With advances in endoscopic, robotic and endovascular techniques, VATS can be performed in a minimally invasive way for managing most pulmonary, pleural and mediastinal diseases [1]. Thoracoscopic pulmonary nodule resection represents the most effective and suitable treatment for pulmonary nodules, and can provide essential histopathological information for definitive diagnosis, staging, and primary treatment [2]. Anesthetic management is essential for successful surgical management. Most thoracoscopic surgeries are performed under general anesthesia with double-lumen endotracheal intubation. Extubation is an essential procedure in anesthesia and critical care medicine. Adverse events at the time of extubation account for a significant proportion of airway management-related serious adverse events [3]. Herein, we presented the case report of a patient whose extubation management was especially challenging.

## Case presentation

**Fig. 1:**
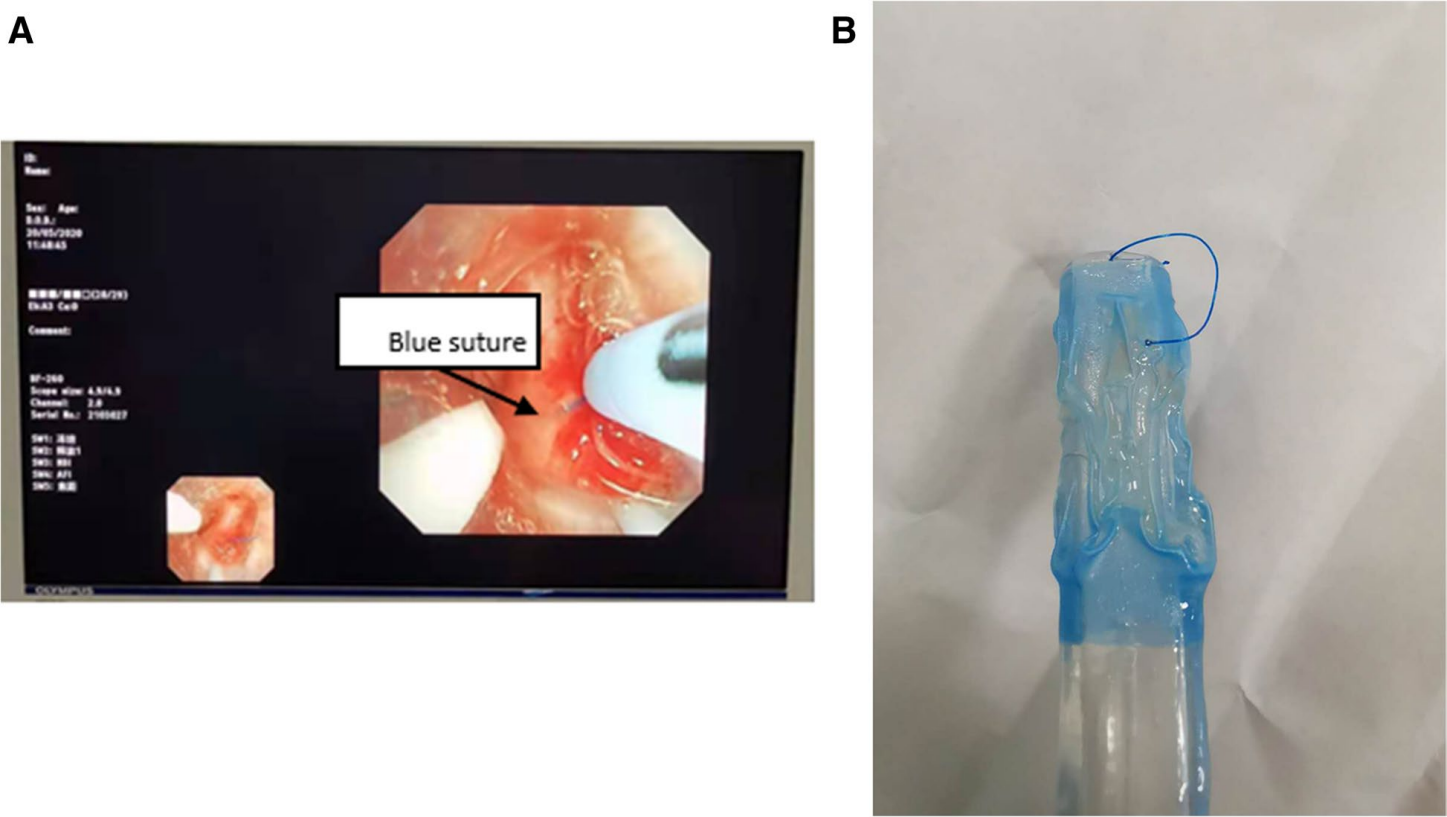
**A** The fiberoptic bronchoscope revealed the surgical suture; **B** The suture stump after the tracheal tube was pulled out

A 48-year-old female, with height of 160 cm and weight of 55 kg, was found to have a left lower lobe nodule in the lung and was scheduled for thoracoscopic wedge resection of the left lower lobe. The anesthesia was induced with 0.04 mg/kg of midazolam, 0.6 μg/kg of sufentanil, 2 mg/kg of propofol and 0.6 mg/kg of rocuronium. Then, a left 35 Fr endobronchial tube (Covidien llc, USA) was successfully intubated at a depth of 28 cm to incisors. Fiberoptic bronchoscopy was used to confirm that there was no problem with the location of the tube after the posture of the patient was changed to the right lateral decubitus position. Anesthesia was maintained with sevoflurane 1.5 vol%, oxygen 2 L/min, remifentanil 250 mcg/hr., 4 mg/kg/h of propofol. The intraoperative pathological diagnosis of the excised mass was invasive adenocarcinoma. Subsequently, the patient underwent VATS combined with left lower lobectomy. The operation time was 1.5 h and the patient was transferred to post-anesthesia care unit (PACU) for decannulation. The patient recovered consciousness from anesthesia after 30 min, exhibiting effective spontaneous ventilation and meeting extubation criteria. Unpredictably, the tracheal tube seemed to be mechanically constrained and we were unable to withdraw the endotracheal tube (ETT). We had to administer propofol and remifentanil intravenously for sedation and checked the airway with a fiberoptic bronchoscope, which revealed that the surgical suture of the bronchial membrane was inserted into the distal end of the left branch of the tracheal tube (Fig. 1A). Thereafter, we held detailed consultations with respiratory physicians and thoracic surgeons. The thoracic surgeon assured that breaking the suture will not cause adverse effects on the patient. It took only 1 s for the respiratory doctor to burn the sutures using an argon electrode (ERBE 20132–177) with the aid of fiberoptic bronchoscope. The mode of the argon electrode was strong electrocoagulation and the power was 35 W. Finally, the bronchial tube was pulled out smoothly (Fig. 1B) and the patient was safely sent back to the ward after CT review, which showed no abnormalities. On the 1, 3, and 7 days after the operation, we followed-up the patient, and there were no related complications such as bronchial leak or bronchial rupture.

## Discussion and conclusions

Extubation is an essential procedure in anesthesia. While safely performed in a large proportion of cases, it can present significant challenges and complications in rare cases [3]. Forceful extubation has been reported to cause vocal cord edema, dislocation of the arytenoid cartilage and laryngeal trauma, which was associated with fatality [4]. Difficulty in extubation due to mechanical issues in the ETT is not uncommon in clinical practice. Previously reported cases of lodged ETTs have been due to failure of cuff deflation, distorted laryngeal anatomy, manufacturing faults, entanglement with feeding tube and the balloon obstructed by a bite block [5]. Unrecognized subglottic stenosis or severe edema physically preventing removal of ETT has been reported [6], as well as an erroneously placed surgical stitch anchoring the ETT to the tracheal wall. Most of these cases have been reported in relation to head and neck surgical procedures [7]. As with our case, Bradley and Sprung reported an unusual case involving the accidental placement of a surgical suture through both the Carlens tube and pulmonary artery [8]. Unfortunately, forceful extubation resulted in massive hemorrhage from the severed pulmonary artery. However, we reported a more fortunate outcome in the current case.

In this case, we discussed in detail with the respiratory doctor and thoracic surgeons whether breaking the sutures would adversely affect the patient and how to break the sutures. Considering that the electrode might damage the tracheal tube, we chose the minimum power that could burn the sutures. Moreover, when the electrode was ready to work, it was necessary to stop the mechanical ventilation, which reduced the oxygen concentration to below 40%.

The learnings from this case were as follows: first, the patient was scheduled for thoracoscopic wedge resection of the left lower lobe. Due to lack of communication with the surgeon in a timely and effective manner, we used 35 Fr left-side endobronchial tube intubation for lung isolation, which caused human interference. It is important to select an appropriate (non-surgical side) tracheal tube. Second, care must be taken even for patients not identified as being at risk of extubation. For example, in this case, pulling out the tracheal tube quickly could lead to the risk of bronchial rupture. Fiberoptic bronchoscopy, as “the third eye of anesthesiologists”, played a clear diagnostic role. Third, the airway manager must be self-aware of potential human factor pitfalls to avoid. Multidisciplinary team training or rounds on adverse airway events might help to improve communication and cooperation for future difficult airway situations that involve multiple specialties.

In conclusion, although difficult decannulation of the airway is a complication seldom encountered, one should always be vigilant when resistance is noted during ETT removal.

## Data Availability

The datasets are available from the corresponding author on request.

## Abbreviations

VATS: Video-assisted thoracoscopic surgery
CT: Chest computed tomography;
PACU: Post-anesthesia care unit
ETT: Endotracheal tube
